# The awareness and acceptance of anti-COVID 19 vaccination in adolescence

**DOI:** 10.1186/s13052-022-01390-8

**Published:** 2022-12-09

**Authors:** Vita Cupertino, Elena Bozzola, Giampaolo De Luca, Emanuela Del Giudice, Giuseppe De Martino, Piero Cannataro, Alberto Eugenio Tozzi, Giovanni Corsello

**Affiliations:** 1 The Adolescent Study Group, The Italian Pediatric Society, Rome, Italy; 2 Cosenza ASP, Cosenza, Italy; 3 Pediatric Unit, Bambino Gesù Children’s Hospital IRCCS, Rome, Italy; 4 Pediatric and Neonatology Unit, Maternal and Child Department, Sapienza University of Rome, Polo Pontino, Latina, Italy; 5 Multifactorial and Complex Diseases Research Area, Bambino Gesù Children’s Hospital IRCCS, Rome, Italy; 6 Department of Health Promotion, Mother and Child Care, Internal Medicine and Medical Specialties “G. D’Alessandro”, University of Palermo, 90100 Palermo, Italy

**Keywords:** Vaccination, COVID-19, Adolescence, Survey

## Abstract

**Background:**

COVID-19 had devastating effects on children’s and adolescents’ life, including neuropsychological impairment, discontinuation of social life and education.

Since June 2021, antiCOVID19 vaccination has become available to adolescents in Italy up to 12 years and since December 2021 to children aged more than 5 years. The pediatric population represents a challenging target for vaccination. Aim of the study is to perform a survey among adolescents to explore factors associated with COVID 19 immunization and their perceptions about COVID-19 vaccines.

**Methods:**

Italian students aged 10–17 years were invited to participate in an anonymous online survey regarding their immunization against COVID-19 and their opinion on the immunization practice through a web link to the questionnaire. The study period was March-June 2022. Statistical analysis was performed with SPSS v 21.

**Results:**

In the study period, 895 students entered the survey. A total of 87.3% of respondents were immunized against SARS-CoV2. The most important predictors of being immunized against SARS-CoV2 were having both parents immunized (*p* < 0, 001) and being aged over 12 years. In the unvaccinated group, the decision was mostly influenced by the family (65.8%). Regardless the immunization status, respondents were willing to receive information about COVID 19 vaccination mostly by their family doctor (51.8%) and at school (28.9%).

**Conclusions:**

Parents’ decisions and attitudes strongly affected the immunization status of adolescents.

Students’ willing to receive COVID 19 vaccine information by family doctors and at school, underline the potential role of paediatricians and school educators in contributing to an increased vaccine coverage among the paediatric age.

**Supplementary Information:**

The online version contains supplementary material available at 10.1186/s13052-022-01390-8.

## Background

COVID-19 had devastating effects on children’s and adolescents’ life, including neuropsychological impairment, discontinuation of social life and education [1]. These effects emerged in Italy during the lockdown period [2]. Safe and effective vaccines against SARS-CoV-2 for children had been available since 2021 and were included in several national immunization strategies [3].

The rationale for children immunization against SARS-CoV-2 is still debated. On one hand, not only vaccines prevent the acute illness, but also protects from long-term sequelae of COVID-19 such as inflammatory multisystem syndrome and long COVID [3]. On the other hand, the risk of severe acute disease in healthy children infected with SARS-CoV- 2 is lower than in adults [4]. Vaccinating children and adolescents against SARS-CoV2 protect from severe disease. Nevertheless, we should keep in mind that the emergence of new variants may decrease the efficacy of the actual vaccines against infection [5].

In Italy, the Ministry of Health recommended as of June 4 and December 7, 2021, extended vaccination for children 12–19 years old and for those 5–11 years of age, respectively.

Nonetheless, as of September 16, 2022, COVID-19 vaccination uptake was 83.55% in adolescents 12–19 years old and only 35,14% in the 5–11 age group [6].

The pediatric population represents a challenging target for vaccination. The extension of the anti- COVID-19 vaccination to children has introduced new and different considerations from a health, social, bio-legal point of view, especially in reference to the active involvement of adolescents.

In fact, despite the existing evidence on safety and effectiveness of COVID-19 vaccines for adolescents and children, many caregivers are still hesitant toward COVID-19 vaccination for their children [5]. Several factors have been associated with hesitancy and/or refusal of pediatric COVID-19 vaccine, including the perception of a low risk of SARS-CoV-2 infection and complications in children as well as the scarcity of studies in children [7]. Moreover, the emergence of Omicron BA.1 variants affected the efficacy of the vaccine in children and adults, rising concerns on immunization strategies, although preventing severe or potentially life-threating disease in the youngest is crucial [4, 8].

It is a common observation that adolescents are socially active, move independently, and are not always willing to follow parental rules. Moreover, it has been found that adolescents in Western Countries behave in a homogeneous manner and like expressing autonomous vaccination opinion [9].

In Italy, any decision regarding medical treatment for children must be made by those who exercise parental responsibility. Nevertheless, starting from the age of 12, children should be consulted in any case. This right also regards minors who are capable of discernment even if younger than 12 years. For these reasons, the approach to any preventive strategy in adolescents is no longer entirely determined by the judgement of parents and pediatricians as in the first few years of life, but is rather the result of a more complex process involving adolescents' thoughts and opinions, their relationship with their parents, friends, doctors, and the information they learn from media [10].

In light of these factors, we performed a survey among adolescents aged 10 to 17 years, which in Italy account for 7,69% of the total population. The scope of the survey is to explore factors associated with COVID 19 immunization and their perceptions on COVID-19 vaccines.

## Materials and methods

### Study design and population

This was a cross-sectional study. Students aged 10–17 years were invited to participate in an anonymous online survey regarding their immunization against COVID-19. In order to conduct the survey, 14 schools distributed in three geographical areas (2 schools from the North, 3 schools from the Center, and 9 schools from the South) were recruited. A web link to the questionnaire was sent to students after obtaining the authorization of the school manager who ensured the intermediation of the teachers according to the internal online channels and methods. The managers of the schools who participated in the research received information on the research methods and objectives beforehand.

Students’ participation was voluntary. Given the simplicity of the questions, all respondents were invited to fill in the questionnaire autonomously, without any influence from teachers. Although the survey was rigorously anonymous, participants had to sign an electronic informed consent prior to filling in the questionnaire. The Italian Society of Bioethics committee approved the survey.

### Questionnaire design

The questionnaire was developed by the Adolescent Study Group of the Italian Paediatric Society in light of the most recent peer-reviewed surveys regarding COVID-19 vaccinations [11].

The questionnaire included the following information (Supplementary File 1):

- personal information such as gender, age, chronic diseases, nationality, usual residence, having siblings, family level of education, work and age;
- opinions about COVID 19 vaccination and the reasons for having been immunized or not; factors that may influence personal’s choice and trustable sources; parents’ immunization status.

### Statistical analysis

The online survey was performed through a Google Forms application. Age was categorized into three groups: 10–11; 12–14; and 15–17. Living residence was grouped into three categories: North (Piedmont, Lombardy, Val d'Aosta, Veneto, Trentino, Friuli-Venezia Giulia, Emilia-Romagna and Liguria); Centre (Tuscany, Lazio, Umbria, Abruzzo, Marche); and South (Molise, Campania, Puglia, Lucania, Calabria, Sicily and Sardinia).

Categorical variables were described as percentages. We performed a logistic regression model to investigate the characteristics associated with COVID immunization of respondents. Adjusted OR from the model we considered significantly associated with the outcome when their 95% confidence interval did not include 1. Statistical analysis was performed with SPSS v 21.

## Results

Between March and June 2022, 904 children viewed the online questionnaire and 895 (99%) accepted to participate in the survey.

Table 1 reports a description of the population included in the study (Table 1).

**Table 1:** General characteristics of the population included in the study (*N* = 895)

		Number	Percent
	Total number of interviews	895	
Sex	Males	392	43,8
	Females	478	53,4
	Unspecificed	25	2,8
Age	10–11 years	91	10,2
	12–14 years	335	37,4
	15–17 years	469	52,4
Residence	Northern Italy	121	13,5
	Central Italy	295	33,0
	Southern Italy	479	53,5
Parents' citizenship	Italian	817	91,3
	Foreign	78	8,7
Siblings	At least 1 sibling	726	81,1
	No siblings	169	18,9
Parents' education level	Both parents with high school diploma or higher	557	62,2
	At least one parent with lower than high school diploma	338	37,8
Parents' employment	Both parents employed	663	74,1
	At least one parent unemployed	232	25,9
Parents' age	Both parents > 45	577	64,5
	At least one parent ≤ 45	318	35,5
Chronic diseases	Yes	61	6,8
	No	834	93,2
Received SARS-CoV2 vaccine	Yes	781	87,3
	No	114	12,7
Parents received SARS-CoV2 vaccine	Both immunized	809	90,4
	One or less immunized	86	9,6

Nearly half of the participants in the study were aged over 14 years whereas those in the 10–11 year were nearly 10%. While there was a majority of participants from Central and Southern Italy, less than 10% had parents with a foreign citizenship. Most parents had an education level higher than high school diploma, most were employed and aged > 45. Nearly 7% of respondents had a chronic disease.

While parents’ age was similar in all geographic areas, some other variations in the sociodemographic level of respondents was observed in different areas: more respondents in Northern Italy had parents with lower education and more respondents in Southern Italy had unemployed parents. The proportion of respondents with chronic diseases varied between 5 and 11% by geographic areas. All these variables were included in the multivariate model to adjust for confounding.

A total of 87.3% of respondents were immunized against SARS-CoV2 and 90.4% of participants had both parents immunized. The immunization rate in foreigner adolescents was similar to Italian ones (79,5% vs 88.0%, respectively).

Immunization rates increased with the age of responders, being 70.3% in the 10-11 year age group, 86.6% in the 12-14 year age group, and 91% in the 15-17 year age group. The proportion of immunized adolescents was similar in all geographic areas. Overall, 60.4% of immunized adolescents received 3 doses of vaccine. The most frequent reason to be immunized was the need to protect the general population (46.9%) followed by the willing to return to a normal life (31.0%). The most important influencers in immunized adolescents were parents and family (69.4%). Among respondents who did not receive the immunization yet, 18.4% declared they are planning to be vaccinated. The most frequent reason for not being immunized was the fear of side effects (40.4%) followed by parent decision to not be immunized (23.7%). A proportion of 14.9% referred that the available vaccine was not efficacious. As in the previous group, not being immunized was largely influenced by parents and family’s decisions (65.8%).

Regardless the immunization status, respondents were willing to receive information about COVID 19 vaccination mostly by their family doctor (51.8%) and at school (28.9%).

The factors associated with immunization of respondents through a multivariable model are reported in Table 2 (Table 2).

**Table 2:** Factors associated with immunization of respondents

Variable		aOR	95% CI	p
Sex		0,988	0,221–4,414	0,987
Age 12–14		4,6	2.3–9,0	< 0,001
Age > 14		13,5	6,1–29.4	< 0,001
Residence Central Italy		1,194	0,504–2,831	0,687
Residence Southern Italy		1,468	0,822–2,623	0,195
Parents' foreign citizenship		1,673	0,719–3,892	0,232
Having siblings		1,103	0,554–2,196	0,781
Parent's higher education level		1,079	0,619–1,883	0,788
At least one parent unemployed		1,047	0,568–1,930	0,883
At least one parent ≤ 45		1,057	0,608–1,838	0,844
Chronic disease		0,642	0,250–1,647	0,357
Parents immunized against SARS-CoV2		90,415	44,512–183,656	< 0,001

Reference category for age: 10–11 years; reference category for residence: Northern Italy

The most important predictor of being immunized against SARS-CoV2 was having both parents immunized. Secondarily, an increasing age was associated with immunization, being children 10–11 years less immunized than older ones. None among the other socio-demographic variables included in the model was associated with vaccination status of participants.

## Discussion

Our study showed a high vaccination rate in Italian adolescents against COVID-19, which increased with the age. These findings are in line with a survey on the willingness to have a COVID-19 vaccination among English students aged 9 -18 years [12]. The different immunization rates by age group may be associate with the timing of recommendations. In fact, The Italian Drug Regulator Agency, AIFA, approved COVID 19 vaccine for 12 to 15-year olds on May 2021, earlier than for children aged less than 11 years. The youngest had the availability of getting their shoot only months later, starting from December 2021. Moreover, in Italy children less than 11 years were exempt from presenting a Green Pass or a Super Green Pass in Italy, required to access many activities and entering gyms swimming pools, indoor restaurants, etc. Finally, even if the Italian Pediatric Society recommend immunization against COVID 19, misleading information on efficacy and safety among the youngest spread [13].

Being immunized was strongly associated with immunization of parents. Immunized adolescents perceived COVID-19 vaccines as a social good and as a mean to return to a normal life. As the survey was anonymous, we did not have the opportunity to discuss the results with the responders. We suppose that the willing to protect the general population is linked to the perception of the risk of a severe COVID-19 infection. In fact, many Italian families experienced relatives hospitalized or dead due to Sars-Cov-2. A Chinese survey revealed that knowing someone diagnosed with COVID-19 may increase the perceived risk of COVID-19 disease and therefore reduce hesitancy in vaccination [14].

On the other side, the fear of side effects was the main reason to withhold with the immunization.

Establishing a communication strategy for COVID-19 vaccination in children and adolescent is challenging. The available literature demonstrates that parents may be hesitant toward immunization. A Chinese survey among parents revealed that even if quite 75% of them was willing to have their children immunized, almost 25% was uncertain or refused COVID 19 vaccination [15]. Another survey reported an even higher proportion of hesitant parents, implying the necessity of comprehensive assessment and health education programs [16, 17].

Although parent hesitancy may affect immunization coverage in children, it is also important to consider the point of view of adolescents on immunization. Even if the vast majority of respondents in our study was immunized, the information provided on their perception about the COVID-19 vaccine may be useful to better understanding the adolescents’ attitudes towards COVID-19 vaccination. Trust in vaccine policy is a significant factor influencing vaccination willingness. In this respect our results are reassuring as adolescents consider immunization against COVID-19 as a tool to reduce the burden of COVID-19. On the other hand, safety of vaccines and their potential side effects are the major concerns that motivate vaccine refusal or hesitancy.

Parents ‘decisions and attitudes strongly affected the immunization status of adolescents although we had no evidence of a contrast between the willingness of respondents to be vaccinated and their family.

Of note, most respondents are willing to receive COVID 19 vaccine information by family doctors and at school, underling the potential role of paediatricians and school educators in contributing to an increased vaccine coverage among adolescents. Our findings also underline that adolescents consider themselves in charge of their own healthcare and are interested in health initiatives, including vaccination. A Swedish survey revealed that one reason for being undecided about the vaccine was that adolescents’ perception they did not know enough about it [18]. Access to more positive information about the safety and effectiveness of adolescent COVID-19 vaccines, either at doctor’s office or at school, may increase the willingness of adolescents to be vaccinated. Indeed, the likeliness of being immunized is associated with many factors, including health care advices, school, community and parents [18, 19].

Increasing vaccine confidence and compliance to immunization schedules within the context of the COVID-19 pandemic is an important end point to eradicate the virus [20].

Our study have limitations. Although respondents were from all geographic areas of Italy, we may have selected those more in favor of immunization against COVID-19 as participation in the study was voluntary. Furthermore, we did not get the opportunity to discuss the answers with the responders and learn more about their perceptions. On the other hand, anonymity may have facilitated more honest feedback from responders as they did not feel judged and were more comfortable in expressing their perceptions. The description of sociodemographic level of respondents by geographic area revealed some differences that may be due to different geographic settings and to selection of respondents. Although we could not access comprehensive information on sociodemographic level of the entire student population, we included sociodemographic variables and having a chronic disease in the multivariate model to adjust for potential confounding.

## Conclusion

Although adolescents mostly perceive vaccines as a social benefit for the general population and a mean for returning to a normal life, doctors' advocacy of COVID-19 vaccines and information provided at school are important sources of reliable vaccine information for them. Increasing communication about the benefits and safety of COVID-19 vaccines for adolescents may increase parents' confidence in COVID-19 vaccines and their willingness to vaccinate their children. Finally, children were more likely to be vaccinated when parents were vaccinated against COVID-19.

## Supplementary Information

**Additional file 1.**

## Data Availability

The Authors confirm that the data supporting the findings of this study are available at the authors ‘office.
