# Supplementary material for: The awareness and acceptance of anti-COVID 19 vaccination in adolescence
Source: Ital J Pediatr. 2022 Dec 9;48:194. doi: 10.1186/s13052-022-01390-8 (PMC9733392; doi:10.1186/s13052-022-01390-8)
Supplement: Supplementary file 1 — Additional file 1. [file 13052_2022_1390_MOESM1_ESM.pdf]

Questionario sulla consapevolezza e l'accettazione della vaccinazione anti- COVID 19 negli adolescenti in Italia (fascia d'età 10-17 anni)

Questa indagine anonima è proposta dal Gruppo di Studio Adolescenza della Società Italiana di Pediatria.

Viene condotta per valutare consapevolezza ed accettazione della vaccinazione anti-COVID19 negli adolescenti.

Rispondere a queste 16 semplici domande richiederà pochi minuti.

Le risposte fornite non consentono l'identificazione del partecipante.

Per ogni domanda è possibile dare solo una risposta.

Se clicchi su "Accetto di continuare e rispondere alle domande" dai il tuo assenso a partecipare all'indagine.
I risultati dell'elaborazione saranno utilizzati esclusivamente per finalità scientifiche.
Il questionario è completamente anonimo. Nessuna risposta consentirà la tua identificazione.

1.
- **a) Accetto di continuare e rispondo alle domande. (vai alla sezione 2 - inizio questionario)**
- **b) Non accetto di continuare (invio modulo)**

2. Genere
- **a)** Femmina
- **b)** Maschio
- **c)** Preferisco non specificare

3. Qual è la tua fascia d'età?
- **a)** 10-11 anni
- **b)** 12-14 anni
- **c)** 15-17 anni

4. In quale Area dell'Italia abiti?
- **a)** Nord (Piemonte, Lombardia, Val d'Aosta, Veneto, Trentino, Friuli-Venezia Giulia, Emilia-Romagna e Liguria)
- **b)** Centro (Toscana, Lazio, Umbria, Abruzzo, Marche)
- **c)** Sud (Molise, Campania, Puglia, Lucania, Calabria, Sicilia e Sardegna)

5. Qual è la tua cittadinanza?
- **a)** Entrambi i tuoi genitori sono italiani
- **b)** Entrambi i tuoi genitori sono stranieri
- **c)** Un genitore è italiano/ un genitore è straniero

5. Hai fratelli/sorelle?
- **a)** No
- **b)** Si uno
- **c)** Sì più di uno

6. Qual è il titolo di studio dei tuoi genitori?
- **a)** Entrambi i tuoi genitori hanno il diploma o la laurea
- **b)** Un tuo genitore ha il diploma o laurea/ l'altro genitore ha la licenza elementare o media
- **c)** Entrambi i tuoi genitori hanno la licenza elementare o media

7. Qual è lo stato lavorativo dei tuoi genitori?
- **a)** Entrambi i tuoi genitori lavorano
- **b)** Solo uno dei tuoi genitori lavora
- **c)** Entrambi i tuoi i genitori sono disoccupati

8. Quanti anni hanno i tuoi genitori?
- **a)** Entrambi i tuoi genitori hanno meno di 45 anni
- **b)** Entrambi i tuoi genitori hanno più di 45 anni
- **c)** Uno dei tuoi genitori ha meno di 45 anni/ l'altro ha più di 45 anni

9. Sei affetto da una malattia cronica?
(diabete, obesità, malattie respiratorie, malattie del sangue, malattie intestinali, malattie degli ormoni, malattie del rene, malattie reumatiche ecc.)
- **a)** Si
- **b)** No

10. **Ti sei vaccinato per il Covid 19?**
    c) Si
    d) No

Se ti sei vaccinato

11. **Se ti sei vaccinato hai fatto:**
    a) 1 dose
    b) 2 dosi
    c) 3 dosi

12. **Se ti sei vaccinato, qual è il motivo principale per cui lo hai deciso?**
    a) Paura di infettarti
    b) Paura di trasmettere l'infezione
    c) Hai avuto un familiare/amico infettato da Covid 19
    d) Hai scelto di vaccinarti come i tuoi amici coetanei
    e) Perché secondo te è necessario fare il vaccino per il bene di tutti
    f) Per tornare alla vita normale come prima della pandemia da Covid 19
    g) È stato deciso dai tuoi genitori
    h) Non volevi vaccinarti ma lo hanno deciso i tuoi genitori

Se non ti sei vaccinato

13. **Se non ti sei ancora vaccinato programmerai la vaccinazione per covid 19 al più presto?**
    a) Si
    b) No

14. **Se non ti sei vaccinato, qual è il motivo principale?**
    a) Non ha ricevuto abbastanza informazioni
    b) Pensi che il vaccino non sia efficace
    c) Hai paura di conseguenze pericolose
    d) Hai avuto difficoltà a prenotare
    e) I tuoi genitori non sono d'accordo sul vaccino
    f) I tuoi genitori non sono d'accordo ma tu vorresti vaccinarti

Sezione finale

15. **Chi ha maggiormente orientato la tua scelta di vaccinarti o di non vaccinarti?**
    a) Medico di famiglia
    b) Famiglia e/o parenti
    c) Amici
    d) Televisione
    e) Social network
    f) Motori di ricerca su internet
    g) Scuola
    h) Farmacista
    i) Associazione

16. **Tramite chi vorresti ricevere informazioni sulla vaccinazione (principalmente)?**
    a) Medico di famiglia
    b) Canali televisivi con programmi dedicati
    c) Giornate dedicate a scuola
    d) Social network

17. **In generale, a parte quella per il Covid19, pensi di aver ricevuto tutte le vaccinazioni, sia quelle obbligatorie sia quelle che vengono consigliate ai bambini e ai i ragazzi?**
    a) Si
    b) No

18. **I tuoi genitori sono vaccinati?**
    a) Si entrambi
    b) No entrambi
    c) Solo uno solo di loro è vaccinato
